# Author Correction: Human activity recognition using magnetic induction-based motion signals and deep recurrent neural networks

**DOI:** 10.1038/s41467-020-16581-2

**Published:** 2020-06-03

**Authors:** Negar Golestani, Mahta Moghaddam

**Affiliations:** Ming Hsieh Department of Electrical and Computer Engineering, University of Southern California, Los Angeles, CA 90089 USA

**Keywords:** Quality of life, Electrical and electronic engineering

Correction to: *Nature Communications* 10.1038/s41467-020-15086-2, published online 25 March 2020.

The original version of this Article contained an error in Fig. 2, in which both Fig. 2a and Fig. 2b were identical. Figure 2a should instead have shown the results for three other activities. This has been corrected in both the PDF and HTML versions of the Article.

